# Impact of Vaccines; Health, Economic and Social Perspectives

**DOI:** 10.3389/fmicb.2020.01526

**Published:** 2020-07-14

**Authors:** Charlene M. C. Rodrigues, Stanley A. Plotkin

**Affiliations:** ^1^Department of Zoology, University of Oxford, Oxford, United Kingdom; ^2^Department of Paediatric Infectious Diseases, St George’s University Hospitals NHS Foundation Trust, London, United Kingdom; ^3^Department of Pediatrics, University of Pennsylvania, Philadelphia, PA, United States

**Keywords:** immunization, vaccines, infectious diseases, infection, children, health economics

## Abstract

In the 20th century, the development, licensing and implementation of vaccines as part of large, systematic immunization programs started to address health inequities that existed globally. However, at the time of writing, access to vaccines that prevent life-threatening infectious diseases remains unequal to all infants, children and adults in the world. This is a problem that many individuals and agencies are working hard to address globally. As clinicians and biomedical scientists we often focus on the health benefits that vaccines provide, in the prevention of ill-health and death from infectious pathogens. Here we discuss the health, economic and social benefits of vaccines that have been identified and studied in recent years, impacting all regions and all age groups. After learning of the emergence of SARS-CoV-2 virus in December 2019, and its potential for global dissemination to cause COVID-19 disease was realized, there was an urgent need to develop vaccines at an unprecedented rate and scale. As we appreciate and quantify the health, economic and social benefits of vaccines and immunization programs to individuals and society, we should endeavor to communicate this to the public and policy makers, for the benefit of endemic, epidemic, and pandemic diseases.

## Introduction

“The impact of vaccination on the health of the world’s peoples is hard to exaggerate. With the exception of safe water, no other modality has had such a major effect on mortality reduction and population growth” (Plotkin and Mortimer, 1988).

**FIGURE 1:**
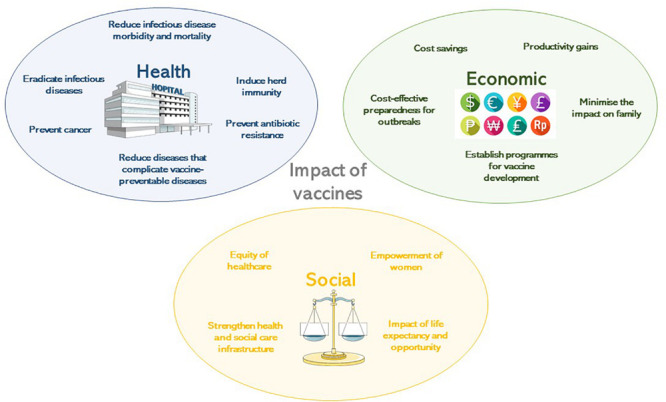
The impact of vaccines according to their health, economic or social benefit.

The development of safe and efficacious vaccination against diseases that cause substantial morbidity and mortality has been one of the foremost scientific advances of the 21st century. Vaccination, along with sanitation and clean drinking water, are public health interventions that are undeniably responsible for improved health outcomes globally. It is estimated that vaccines have prevented 6 million deaths from vaccine-preventable diseases annually (Ehreth, 2003). By 2055, the earth’s population is estimated to reach almost 10 billion (United Nations Department of Economic and Social Affairs, 2019), a feat that in part is due to effective vaccines that prevent disease and prolong life expectancy across all continents. That said, there is still much to be done to ensure the financing, provision, distribution, and administration of vaccines to all populations, in particular those which are difficult to reach, including those skeptical about their protective value and those living in civil disruption. Agencies including the World Health Organization (WHO), United Nations Children’s Fund (UNICEF), Gavi, the Vaccine Alliance, The Bill & Melinda Gates Foundation, and the Coalition for Epidemic Preparedness Initiative (CEPI), with their multiple funding streams have been instrumental in expanding vaccine benefits to all. These importance of these organizations in global co-operation and participation was essential in the setting of the 2019 global pandemic of SARS-CoV-2, in light of the health and economic impact of COVID-19 on societies in high-, middle- and low-income countries. This review will highlight the benefits of vaccinations to society from the perspectives of health, economy, and social fabric (Figure 1), which need to be considered in the overall assessment of impact to ensure that vaccines are prioritized by those making funding decisions.

## Brief History of Vaccine Development

Human use of preparations to prevent specific infections have been described since 1500 AD, beginning in China (Needham, 2000) where smallpox was prevented by variolation, which is the introduction of material from scabs into the skin. In 1796 in the United Kingdom, Edward Jenner observed the immunity to smallpox of milkmaids having previously had natural infection with cowpox (Jenner, 1798). He determined that inoculating small amounts of pus from the lesions of cowpox, presumably containing a virus related to vaccinia, into susceptible hosts rendered them immune to smallpox. The vaccine against smallpox was developed in 1798. The next phase of scientific developments involving the manipulation of infectious agents to extract suitable vaccine antigens took almost a century of research. Louis Pasteur’s work with attenuation by oxygen or heat led to live-attenuated chicken cholera, inactivated anthrax and live-attenuated rabies vaccines at the turn of the 20th century (Pasteur, 1880, 1881, 1885). Alternative methods of attenuation using serial passage of *Mycobacterium bovis* led to the live Bacille Calmette-Guerin (BCG) (Calmette, 1927) vaccine, still in use today for the prevention of tuberculosis. Serial passage was also used in the development of yellow fever vaccines (Theiler and Smith, 1937a) which are grown in chicken embryo tissues (Theiler and Smith, 1937b). Whole cell killed bacterial vaccines were developed when methods to treat and kill bacteria through heat or chemicals were established and whole cell typhoid, cholera and pertussis vaccines resulted at the end of the 19th Century. In 1923, Alexander Glenny and Barbara Hopkins developed methods to inactivate bacterial toxins with formaldehyde, leading to the diphtheria and tetanus toxoid vaccines (Glenny and Hopkins, 1923).

Advances in virus culture *in vitro* allowed viral pathogens to be studied in greater detail and attenuation methods due to cultivation in artificial conditions led to the live oral polio, measles, rubella, mumps and varicella virus vaccines. In the 1960’s at the Walter Reed Army Institute of Research, vaccines were developed using capsular polysaccharides (Gold and Artenstein, 1971; Artenstein, 1975), of encapsulated organisms including meningococci and later pneumococci (Austrian, 1989) and *Haemophilus influenzae* type b (Hib) (Anderson et al., 1972). To protect against multiple serotype variants of polysaccharide capsules, polyvalent vaccines were developed and later conjugated to carrier proteins to enhance their efficacy in infants in particular by recruiting T-cell mediated help to induce memory B-cells (Schneerson et al., 1980). Vaccines made solely from proteins were rare, with the exception of the toxoid vaccines, but the acellular pertussis vaccine containing five protein antigens, was developed to mitigate the unwanted effects of the whole cell vaccine (Sato and Sato, 1999).

The end of the 20th century marked a revolution in molecular biology and provided insights into microbiology and immunology allowing a greater understanding of pathogen epitopes and host responses to vaccination. Molecular genetics and genome sequencing has enabled the development of vaccines against RNA viruses possessing multiple variants of epitopes, such as the live and inactivated influenza vaccines (Maassab and DeBorde, 1985) and live rotavirus vaccines (Clark et al., 2006). DNA manipulation and excision allowed the use of surface antigen for hepatitis B viral vectors (Plotkin, 2014). The human papilloma virus (HPV) vaccine benefits from enhanced immunogenicity due to the formation of virus-like particles by the L1 antigen of each virus contained in the vaccine (Kirnbauer et al., 1992). Bacterial genome sequencing has provided in depth analysis of meningococcal antigens, to identify potential proteins for meningococcal B vaccines (Serruto et al., 2012).

Vaccine development was tested in 2020 when a novel coronavirus, SARS-CoV-2, emerged from China causing a severe acute respiratory illness, which subsequently spread globally. Within 5 months of the discovery of this virus (7th January 2020) (Zhu et al., 2020) and person-person transmission (Chan et al., 2020), 5,697,334 cases had been identified, with orders of magnitude likely not measured and almost no country escaped the pandemic. Owing to the previous advances in vaccinology, by 8th April 2020, there were 73 vaccine candidates under pre-clinical investigation (Thanh Le et al., 2020). Of these, six were in Phase 1 or 1/2 trials and one was in Phase 2/3 trials by 28th May 2020. The rapidity of this response demonstrated the ability to harness existing technologies including: RNA vaccine platforms (NCT04283461), DNA vaccine platforms (NCT04336410), recombinant vector vaccines (NCT04313127, NCT04324606) and adjuvants. The regulation, manufacturer and distribution of these vaccines will require expedition given the global public health need, from a period of many years to a matter of months. The efficacy and health impact of these vaccines is yet to be established, but if they are effective, then vaccines need to be made available for all global regions affected by SARS-CoV-2. The funding of this endeavor will prove challenging in a global context of national social and economic lockdown and massive government borrowing, but the justification for this provision will be through the multiple benefits to society that will need healthy citizens to rebuild economies in the decades post-COVID-19.

The history of vaccination is not complete without describing the public health intervention that led to the routine use of these vaccines for children globally. The Expanded Program of Immunization (EPI) was founded by WHO in 1974 with the aim of providing routine vaccines to all children by 1990 (World Health Assembly, 1974). In 1977, global policies for immunization against diphtheria, pertussis, tetanus, measles, polio, and tuberculosis were set out. The EPI includes hepatitis B, Hib, and pneumococcal vaccines in many areas and by 2017, 85% of the world’s children (12–23 months of age) received diphtheria, pertussis, tetanus, and measles vaccines (World Bank, 2019).

## Health Benefits of Vaccination

### Reduction in Infectious Diseases Morbidity and Mortality

The most significant impact of vaccines has been to prevent morbidity and mortality from serious infections that disproportionately affect children. Vaccines are estimated to prevent almost six million deaths/year and to save 386 million life years and 96 million disability-adjusted life years (DALYs) globally (Ehreth, 2003). The traditional measures of vaccine impact include: vaccine efficacy, the direct protection offered to a vaccinated group under optimal conditions e.g., trial settings; or vaccine effectiveness, the direct and indirect effect of vaccines on the population in a real-life setting (Wilder-Smith et al., 2017). Providing a numerical measure of vaccine impact therefore involves estimating the extent of morbidity and mortality prevented. In the United States in 2009, amongst an annual birth cohort vaccinated against 13 diseases it was estimated that nearly 20 million cases of disease and ∼42,000 deaths were prevented (Zhou et al., 2009). Infectious diseases that accounted for major mortality and morbidity in the early 20th century in the United States all showed over a 90% decline in incidence by 2017 from the pre-vaccine peak incidence (Roush and Murphy, 2007), due to high vaccine uptake of over 90% for the DTaP (diphtheria, tetanus, and acellular pertussis), MMR (measles, mumps, and rubella) and polio vaccines (World Health Organisation, 2019a; Table 1). A similar pattern of infectious diseases reduction was seen across other high-income countries, demonstrating the efficacy of vaccines when available and accessible.

**TABLE 1:** Vaccine impact in United States comparing the incidence of diseases prior to the implementation of vaccine (Roush and Murphy, 2007), described as the pre-vaccine era and the vaccine coverage (Hill et al., 2017) and disease incidence (Centers for Disease Control and Prevention, 2017) in 2017, as reported by the Centers for Disease Control and Prevention.

**Vaccine**	**Peak cases in prevaccine era**	**Vaccine coverage in children 19–35**	**Cases in 2017**	**Disease reduction**
	**(year)**	**months old (% [95% Cl])**	**(*n*)**	**(%)**
Smallpox	110,672(1920)	−	0	100
Diphtheria	30,508(1936)	94.0(93.3−94.7)	0	100
Measles (non-imported)	763,094(1958)	91.5(90.6−92.3)	99	99.99
Mumps	212,932(1964)	91.5(90.6−92.3)	6,109	97.13
Rubella	488,796(1964)	91.5(90.6−92.3)	7	100.00
Congenital rubella syndrome	20,000(1964−65)	−	5	99.98
Pertussis	265,269(1934)	94.0(93.3−94.7)	18,975	92.85
Polio (paralytic)	21,269(1952)	92.7(91.9−93.5)	0	100
Tetanus	601 (1948)	94.0(93.3−94.7)	33	94.51

**FIGURE 2:**
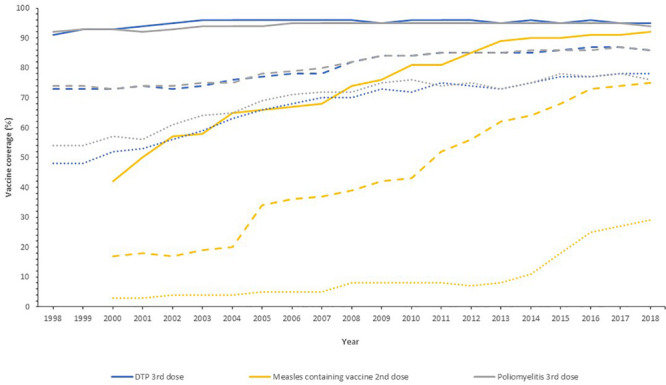
Vaccine uptake across different regions defined by economic status by the World Bank into high- (solid line), middle- (dashed line), and low-income countries (dotted line) for the past 20 years. Data from the World Health Organization and UNICEF dataset “Coverage Estimates Series” (World Health Organization [WHO] and United Nations Children’s Fund [UNICEF], 2019).

**FIGURE 3:**
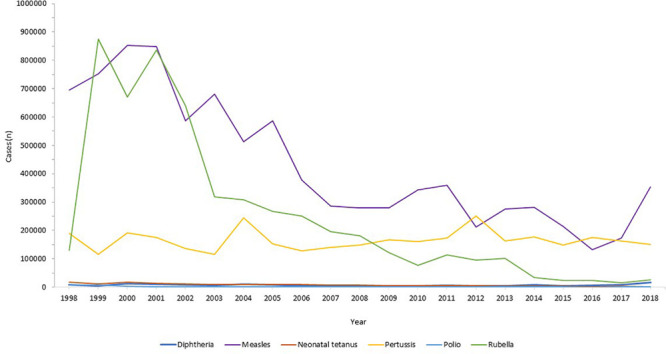
Reduction in infectious diseases globally. Across all world regions, data from the WHO, for the last 20 years showing the control of diphtheria and tetanus and the decline in rubella and congenital rubella syndrome (data not shown). Data from the World Health Organization dataset “Reported cases of vaccine-preventable diseases” (World Health Organisation, 2019c).

Globally, the provision of vaccines is more challenging in many low- and middle- income countries (LMIC), as evidenced by the failure to make the EPI vaccines available to every child by 1990, irrespective of setting (Keja et al., 1988). Central to this is limited financial resources, but other barriers to vaccine introduction include: underappreciation of the value of vaccines locally/regionally though insufficient relevant data on disease burden, vaccine efficacy, or cost-effectiveness; inadequate healthcare infrastructure for vaccine handling, storage, programmatic management, and disease surveillance; and lack of global, regional or local policy-making and leadership (Munira and Fritzen, 2007; Hajjeh, 2011). In 2018, the global uptake of three doses of DTaP reached 86% which corresponded to 116,300,000 infants (World Health Organisation, 2019a). The vaccine coverage is, however, variable between low-, middle- and high-income countries because of a combination of economic and political circumstances as well as variable access to non-governmental support from Gavi, the Vaccine Alliance (Turner et al., 2018; Figure 2). Nevertheless, there has been a decrease in the global burden of diseases caused by vaccine-preventable pathogens (Figure 3) enabling healthier lives for many millions of children. A further benefit following vaccination, is the evidence that although vaccines may not always prevent an infection, for example VZV or pertussis, a milder disease course may follow (Andre et al., 2008; Bonanni et al., 2015).

### Eradication of Infectious Diseases

Global disease eradication can be achieved for pathogens that are restricted to human reservoirs. For eradication of infectious diseases, high levels of population immunity are required globally, to ensure no ongoing transmission in our well-connected world (Andre et al., 2008). Furthermore, surveillance systems must be in place to monitor the decline in disease, with accurate and reliable diagnostic testing to monitor ongoing cases. At the time of writing, the only infectious disease that has been eradicated in humans by vaccination is smallpox. This disease had afflicted humans for millenia, with the earliest evidence found in Egyptian mummies from 1000 BC (Geddes, 2006). Jenner’s successful development of the smallpox vaccine using vaccinia virus (Jenner, 1798) led to the ultimate eradication of the disease through ring vaccination as announced by the World Health Assembly in 1980 (Strassburg, 1982), which was an historic public health achievement. The second example of eradication was of the rinderpest virus in livestock, an infection that indirectly led to human loss of life through loss of agriculture leading to humanitarian crises through famine and poverty. Rinderpest virus infects cattle, buffalo and numerous other domestic species, with widespread disease affecting large parts of Africa and Europe in the 19th century (Roeder et al., 2013). The Plowright tissue culture rinderpest vaccine, developed during the 1950s, was used for mass vaccination campaigns, alongside other public health measures, leading to eradication in 2011 (Morens et al., 2011).

The next infection targeted for eradication is wild polio virus. This devastating paralytic disease routinely afflicted children and adults in both industrialized and developing settings, prior to the development of vaccines. Two polio vaccines, the inactivated polio vaccine (IPV) and the live-attenuated oral polio vaccine (OPV) became available in 1955 and 1963, respectively (Plotkin, 2014), both able to protect against all three wild types of polio virus. Both vaccines have been used globally, with live-attenuated OPV much cheaper and easier to administer but carrying the risk of causing circulating vaccine-derived poliovirus (cVDPV) owing to back-mutation and re-acquisition of neurovirulence. Hence, due to its safety IPV was preferred in industrialized regions and those where the polio incidence was low. In 1998, the Global Polio Eradication Initiative, the largest public-private partnership led by national governments in partnership with the WHO, Rotary International, United States Centers for Disease Control and Prevention (CDC), and UNICEF was launched with the aim of global polio eradication by 2000. Although this target was not met due to lack of funding, political will, and competing health initiatives, there was a 99% reduction in polio incidence by 2000 (Lien and Heymann, 2013). By 2003, there were only six endemic countries with new cases: Egypt, Niger, India, Nigeria, Afghanistan, and Pakistan, of which only the latter four had new cases by 2005. Eradication in India was problematic due to the high birth rates and poor sanitation amongst densely populated regions with marginalized communities and high population mobility (Thacker et al., 2016). India was declared polio free in 2014. Wild polio virus type 2 was eradicated in 2015, the last case of wild type 3 was in 2012 and eradication announced in 2019, with wild type 1 virus remaining in two countries, Pakistan and Afghanistan (World Health Organisation, 2019b). In 2019, Nigeria was declared 3 years free of wild polio, the last country in Africa to declare any cases. In the first 6 months of 2020, there were 51 and 17 cases of wild type 1 polio reported in Pakistan and Afghanistan respectively (Global Polio Eradication Initiative, 2019). Ongoing programs to roll out universal vaccination in both countries remain hindered by armed conflict, political instability, remote communities and underdeveloped infrastructure. The risk of the OPV recipients developing cVDPV disease, with transmission through the faeco-oral route to cause outbreaks of vaccine-derived paralytic poliomyelitis remains a concerning obstacle in the eradication process, requiring intensive surveillance.

### Herd Immunity

The overriding health benefit perceived by most vaccine recipients is their personal, direct, protection. The added value of vaccination, on a population level, is the potential to generate herd immunity. Where a sufficiently high proportion of the population are vaccinated, transmission of the infecting agent is halted thereby protecting the unvaccinated, who may be those too young, too vulnerable, or too immunosuppressed to receive vaccines. Highly successful vaccination programs have been in place as part of the routine EPI, against encapsulated bacteria that are carried asymptomatically in the oropharynx but that can invade and cause septicemia and meningitis in all age groups. Vaccines against *Neisseria meningitidis* (Gold and Artenstein, 1971), *Streptococcus pneumoniae* (Austrian, 1989), and Hib (Anderson et al., 1972) were developed in the 1960s, 1970s, and 1980s, respectively, using their polysaccharide capsules as vaccine antigens, which successfully induced protective immunity (direct protection). Conjugation of these polysaccharides to carrier proteins in the 1990s improved their efficacy by not only ensuring a T cell response and immune memory, but by reducing acquisition of pharyngeal carriage of these organisms, thus providing indirect protection and thereby preventing ongoing transmission (Pollard et al., 2009). This was first observed in national carriage studies in the United Kingdom in 1999–2001 during a mass vaccination campaign against serogroup C *N. meningitidis* (Maiden et al., 2008) and was a major contributing factor to the declining disease thereafter.

Herd (population) immunity requires high levels of vaccine uptake, to limit the number of unvaccinated people and the opportunity for pathogen transmission between them. The proportion of a given population required to induce herd immunity through vaccination is lower for the bacterial infections and conjugate polysaccharide vaccines, as their basic reproductive number (R_0_) is lower than viral infections like measles, varicella or polio (Table 2). Measles virus can cause devastating disease ranging from acute presentations with pneumonia or encephalitis, to immune amnesia and long-term complications such as subacute sclerosing panencephalitis (Mina et al., 2015, 2019; Moss, 2017; Petrova et al., 2019). The live-attenuated measles vaccine is highly efficacious and the first dose is recommended at 9–12 months of age. To protect those who cannot receive live vaccines (younger infants, pregnant women, the immunosuppressed) from acquiring measles in the community, at least 93–95% of the population is required to be vaccinated with two doses in order to interrupt measles virus transmission. In many countries in Europe and in the United States, this level of vaccination uptake is falling (Wise, 2018), due to a combination of reduced accessibility to health services and vaccine misinformation. As a result, some countries, including the United Kingdom and United States, where elimination of measles had been declared have had a resurgence of disease (Wise, 2019). For high-risk individuals who are unable to be vaccinated, herd immunity represents a life-saving protection strategy against many infections. An alternative strategy, cocooning, has been employed with limited success for pertussis and influenza (Grizas et al., 2012), where their close/household contacts are vaccinated to prevent transmission.

**TABLE 2:** Vaccines with the potential to induce herd immunity, with the infectious agent, vaccine type, and thresholds of population vaccination needed for herd immunity (Peltola et al., 1999; Whitney et al., 2003; Donaghy et al., 2006; Fine and Griffiths, 2007; Maiden et al., 2008; Curns et al., 2010; Paulke-Korinek et al., 2011; Plans-Rubio, 2012; Daugla et al., 2014; Tabrizi et al., 2014; Funk et al., 2019; Palmer et al., 2019).

**Disease**	**Vaccine**	**Herd immunity mechanism**	***%* of population requiring immunization to achieve herd immunity**	**References**
*Bacteria*				
*Neisseria meningitidis*	Conjugated polysaccharide vaccines against A,C, W,Y	Reduced acquisition of oropharyngeal carriage of hypervirulent (genotypic and/or phenotypic) strains	17–26	Maiden et al., 2008; Daugla et al., 2014
*Streptococcus pneumoniae*	Conjugated polysaccharide vaccines 7-, 10-, 13-valent	Reduced acquisition of oropharyngeal carriage of hypervirulent (genotypic and/or phenotypic) strains		Whitney et al., 2003
*Haemophilus influenzae* type b	Conjugated polysaccharide vaccines against type b	Reduced acquisition of oropharyngeal carriage of hypervirulent (genotypic and/or phenotypic) strains	50	Peltola et al., 1999
*Viruses*				
Measles virus	Live-attenuated measles virus	Reduced contact/airborne transmission of the virus, for a disease where the R_0_ > 1 (estimated 11–18).	91–95	Funk et al., 2019
Mumps virus	Live-attenuated mumps virus	Reduced airborne transmission of the virus, for a disease where the R_0_ > 1 (estimated 7–14).	75–86	Donaghy et al., 2006
Rubella virus	Live-attenuated rubella virus	Reduced contact/airborne/vertical transmission of the virus, for a disease where the R_0_ > 1 (estimated 6–14).	83–94	Plans-Rubio, 2012
Varicella virus	Live-attenuated varicella zoster virus	Reduced contact/airborne transmission of the virus, for a disease where the R_0_ > 1 (estimated 7–11).	86–91	Plans-Rubio, 2012
Wild-type polio virus	Live, oral polio vaccine	Reduced faeco-oral transmission of the virus, for a disease where the R_0_ > 1 (estimated 5–7).	80–86	Fine and Griffiths, 2007
Rotavirus	Live, multivalent rotavirus vaccine	Reduced faeco-oral transmission of the virus, for a disease where the R_0_ > 1 (estimated 16–25).	>70	Curns et al., 2010; Paulke-Korinek et al., 2011
Human papilloma virus	Bivalent vaccines with serotype 16, 18	Reduced transmission of the virus between sexual partners.	20 with girls only vaccinated	Tabrizi et al., 2014; Palmer et al., 2019

Herd immunity has been observed for gastrointestinal infections with vaccines against cholera (oral cholera vaccine) and rotavirus (oral rotavirus vaccines). Early adopters of rotavirus vaccines included the United States (2006) and Austria (2007) where there were dramatic reductions in disease observed in the vaccinated infant cohort, and also in the older age groups of children and adults (Curns et al., 2010; Paulke-Korinek et al., 2011), suggesting that the reduction in disease and shedding of virus in the stool stopped transmission to healthy household contacts. For the OPV, herd protection may also be induced through vaccine virus shedding and spread to unvaccinated people (Fine and Griffiths, 2007).

### Reduction in Secondary Infections That Complicate Vaccine-Preventable Diseases

Vaccines can prevent diseases beyond the specific infection they are designed to target. Infections with pathogens, in particular viruses, can predispose to the acquisition of other bacterial infections. For example, influenza virus infection, both seasonal and pandemic, is frequently complicated by bacterial pneumonia and acute otitis media (OM), and infrequently *Aspergillus* pneumonia/pneumonitis. During the influenza pandemic of 1918–19, secondary bacterial bronchopneumonia with *S. pneumoniae, Streptococcus pyogenes*, *H. influenzae*, and *Staphylococcus aureus* identified at autopsy, likely contributed to the excess mortality observed amongst healthy children and adults (Morens and Fauci, 2007). Influenza vaccinations can be beneficial in preventing these complications and also morbidity including acute OM in children; a systematic review demonstrated influenza vaccine efficacy against OM of 51% (21–70%) (Manzoli et al., 2007). Further, there is evidence that inactivated influenza vaccines administered to pregnant women can reduce the hospital admission with acute respiratory illnesses in their infants up to 6 months of age (Regan et al., 2016). Amongst pregnant, HIV-negative women in South Africa, infants (<3 months) were protected against hospitalization with all-cause lower respiratory tract infections with a vaccine efficacy of 43% (*p* = 0.05), including primary viral and secondary bacterial causes (Nunes et al., 2017). Additionally, in children pneumococcal conjugate vaccines were observed to reduce the incidence of influenza-associated hospital admissions in United States (Simonsen et al., 2011), Spain (Dominguez et al., 2013), and South Africa (Madhi et al., 2004; Abadom et al., 2016), through the prevention of secondary bacterial infections following primary influenza infection.

The introduction of the live-attenuated measles vaccine in the 1970s was observed to reduce both measles and non-measles mortality in children (Aaby et al., 2003). Measles causes severe pneumonia, encephalitis, and the long-term sequel of subacute sclerosing panencephalitis (Moss, 2017), but the decline in mortality was not limited to preventing these alone (Aaby et al., 2003). Mathematical modeling of vaccination and immunological research demonstrated that measles causes an immunological amnesia, eliminating B cell populations and thus immune memory, leaving measles survivors susceptible to all the infective agents they had previously developed immunity against; it is estimated to take 3 years for immune recovery to occur (Mina et al., 2015).

### Prevention of Cancer

Historically, vaccines were developed against very severe infections with major morbidity and mortality from acute disease. As non-communicable diseases, including cancer, become the most frequent causes of death in industrialized countries and some developing countries, vaccines are being used to prevent these too, when the infectious agents are involved in carcinogenesis. Hepatitis B prevalence is high in regions of East Asia, sub-Saharan Africa, and the Pacific Islands. Chronic hepatitis B infection can lead to liver cirrhosis and hepatocellular carcinoma (Bogler et al., 2018). Vertical transmission of hepatitis B is problematic as 70–90% of babies born to HbsAg and HbeAg positive mothers will become infected without prophylaxis administered to babies; with ∼90% of infants developing chronic hepatitis (Borgia et al., 2012; Gentile and Borgia, 2014). The chronic hepatitis B carriage status of mothers is routinely checked at the start of pregnancy, in order to assess the need to vaccinate the infant after birth. The use of both hepatitis B vaccine, containing hepatitis B surface antigen, and immunoglobulin containing hepatitis B antibody can be used to minimize vertical transmission, with evidence from a 20-year-long study in Thailand demonstrating 100% prevention of transmission (Poovorawan et al., 2011).

The sexually transmitted HPV is responsible for genital tract and oropharyngeal infections as a precursor to causing oncological disease affecting the cervix, vagina, vulva, penis, anal tract, and pharynx in both men and women. Cervical cancer is the fourth most common cancer globally, with 528,000 new cases annually and peak incidence in young women aged 25–34 years (Ferlay et al., 2012). The HPV serotypes 16 and 18 carry a high-risk for cervical cancer (Wang et al., 2018) and vaccination against these specific serotypes has been available since 2006 through bivalent (16 and 18), quadrivalent (6, 11, 16, and 18), and nonavalent (6, 11, 16, 18, 31, 33, 45, 52, 58) vaccines, which are now available to individuals from the age of 9 years (Gupta et al., 2017). A vaccination program started in the United Kingdom in 2008, and at the time of writing over 10.5 million doses had been given to girls (Public Health England, 2018), with the aim of preventing primary infection with HPV. The vaccine coverage was 83.8% for 13–14 year old girls in England in 2017/18 (Public Health England, 2019). In July 2018, the vaccine was approved for use in boys (Public Health England, 2019). After a decade of use, there has been an observed decline in the genital infections caused by serotypes 16 and 18 (Public Health England, 2018), with further time needed to observe the fall in cervical cancer incidence. However, the incidence of pre-invasive cervical diseases has been reduced by 79–89% in Scottish women over 20 who were vaccinated with bivalent HPV vaccine when aged 12–13 years, with evidence of herd protection (Palmer et al., 2019), offering a promising outlook for the reduction of cervical cancer in the future. An additional benefit of HPV vaccines, is their impact on neonatal morbidity and mortality, through the reduction in surgical treatment of cervical neoplasias, and the related preterm births and complications (Soergel et al., 2012).

### Preventing Antibiotic Resistance

The rise in antimicrobial resistance (AMR) is a universal threat. The use of antibiotics in humans, exposes the bacteria that reside in our microbiota to selection pressures resulting in the development of AMR. As the bacteria constituting the host microbiota are frequently responsible for invasive diseases such as: meningitis, pneumonia, urinary tract, or abdominal infections, the risk of developing infections that are difficult or eventually impossible to treat is fast becoming a reality (Brinkac et al., 2017). In regions where resistant pathogens are circulating at high frequency, such as India or regions of Europe (Logan and Weinstein, 2017), patients will be faced with choosing between having elective surgical procedures or chemotherapy for malignancy, and the risk of acquiring potentially untreatable, multi-drug resistant bacterial infections (Liu et al., 2016). Vaccination is crucial in mitigating this risk, by preventing people from developing viral and bacterial infections in the first instance, and therefore reducing the antibiotic burden to which their microbiota are exposed. The development of AMR in bacteria is a cumulative process with frequent, repeated exposure to broad spectrum antibiotics as a major driver. Children and the elderly who are at particular risk of infection can benefit from vaccines against common primary and secondary infections such as: pneumonia (prevented by PCV, PPSV, influenza, and measles vaccines), OM (PCV, Hib, and measles vaccines), cellulitis secondary to VZV (VZV vaccine), and typhoid fever (typhoid vaccine) which alleviates the need for antibiotics being prescribed or bought (Kyaw et al., 2006; Palmu et al., 2014). The extent to which vaccination contributes to antimicrobial stewardship was highlighted by its inclusion in vaccine cost-effectiveness analyses as part of national United Kingdom policy (Bonanni et al., 2015).

## Economic Benefits

### Cost Savings

Vaccines are highly beneficial on a population level and also cost-effective (Shearley, 1999) in comparison to other public health interventions (Bloom et al., 2005). Government departments are required to perform systematic economic analyses of vaccines and vaccine programs to justify their purchase in view of pressure on public and private finances globally, this was exacerbated by the 2008 financial crash. A vaccination program has clear direct costs including: vaccine purchase, infrastructure to run the program and maintain the cold chain, and healthcare/administration personnel. Governments, sometimes supported by charities and non-governmental organizations, invest in these with the intention of improving health. The reduction in morbidity and mortality associated with successful vaccine programs, through a combination of direct and indirect protection, has led to reduced incidence of diseases and their associated treatments and healthcare costs (Deogaonkar et al., 2012). This potentially leads to economic growth, with less money spent owing to the costs averted through fewer medical tests, procedures, treatments and less time off work by patients/parents. Additionally, the use of combination vaccines e.g., DTaP/IPV/Hib/HepB provides protection against an increased number of diseases, with no additional infrastructure costs i.e. the same number of injections per child within existing immunization programs.

The cost-effectiveness analyses of vaccination programs demonstrate that they are overwhelmingly worth the investment, with most programs costing less than $50 per life gained, orders of magnitude less than prevention of diseases like hypertension (Ehreth, 2003; Bloom et al., 2005). The returns on investment in vaccines, given their increasing provision through Gavi, have been estimated at 12–18% (Bloom et al., 2005), but this is likely an underestimate. The monetary advantages of vaccination programs are important both to industrialized nations, such as the United States which obtains a net economic benefit of $69 billion, but also in 94 LMIC where investment of $34 billion, resulted in savings of $586 billion from the direct illness costs (Ozawa et al., 2016; Orenstein and Ahmed, 2017). The net economic impact of eradication of disease has been estimated for both smallpox and polio. For smallpox, the eradication costs were over 100 million USD, but there are cost savings of 1.35 billion USD annually, with elimination of polio estimated to save 1.5 billion USD annually (Barrett, 2004; Bloom et al., 2005). A less well-considered economic saving, not captured in cost-effectiveness or cost-benefit analyses, is from the prevention of long-term morbidity following acute infections (Bloom et al., 2005), for example hearing impairment following pneumococcal meningitis or limb amputation following meningococcal disease, along with broader productivity gains (Deogaonkar et al., 2012), which could have a major impact on LMIC adoption of vaccine programs.

### Productivity Gains

The relationship between health and the economy is bidirectional, whereby economic growth enables funding in investments that improve health; and a healthy population contributes to and enhances an economy. These benefits of vaccinations and other public health interventions including sanitation, clean water, and antibiotics, are important for social as well as economic reasons. It has been suggested that the economic impact of vaccines should be considered more broadly than just the averted healthcare costs from prevented illness episodes and associated carer costs (Deogaonkar et al., 2012; Barnighausen et al., 2014; Bonanni et al., 2015; Gessner et al., 2017; Wilder-Smith et al., 2017). Bärnighausen et al. (2011), set out a framework to consider productivity gains measured by: outcome and behavior; community health and economic externalities; risk reduction; and health gains. Healthy children demonstrate improved educational attainment at school through better attendance and better cognitive performance (Barham and Calimeria, 2008; Bloom et al., 2011; Deogaonkar et al., 2012). The impact of hearing loss from mumps, rubella or pneumococcal infections, or visual impairment from measles may require specific educational support, whereas the cognitive deficits from those childhood infections may require substantial remedial input. As more children survive to adulthood, a larger adult workforce is available, who when healthy can work for longer and more productively both physically and mentally (Bloom and Canning, 2000; Bloom et al., 2005); though to date this has been observed largely following other health improvements, not vaccination specifically (Jit et al., 2015). As a result of vaccination healthy and economically successful populations have lower fertility rates and smaller families (Sah, 1991; Andre et al., 2008). With improved health and therefore life expectancy, there is a wider effect on families who may choose to invest more money in their future, for example to enhance their education or through savings (Jit et al., 2015). Overall, vaccine programs should be viewed as an investment in human capital, providing enduring impact on economies worldwide.

### Minimizing the Impact on Family

The economic impact of adult illness is evident from loss of productivity and pay for the duration of the illness and recovery period. The impact of childhood illness falls primarily on their adult carers, generally parents. In most industrialized regions, two-parent families are reliant on both parents undertaking at least part-time or full-time work. Therefore, when a child is unwell with childhood illnesses, which may or may not necessitate admission to hospital, the parent will invariably have to forego their paid employment to care for the child. In seven European countries one parent or carer required time off work in 39–91% of rotavirus gastroenteritis cases (Van der Wielen et al., 2010). This loss of productivity in the parental workforce tends to disproportionately affect women, but loss of either parental attendance at work reduces overall employer productivity and in the short-term is rarely replaced. This argument was made for the impact of chicken pox on children, whereby the exclusion from school mandates parental caring at home for a period until the lesions are crusted over. VZV vaccines are estimated to have had a similar impact as rotavirus vaccine in United States studies (Lieu et al., 1994). In many regions, mothers are still the primary carers, spending their days at home caring for children and maintaining the household; in these settings, the impact on this unpaid work is harder to determine.

It is of paramount importance to quantify and include productivity gains and the wider effects in analyses of impact for vaccines with only moderate efficacy, as calculated using traditional metrics. Vaccines such as the RTS,S/AS01 malaria vaccine, CYD-TDV dengue vaccine and rotavirus vaccine used in LMIC all have limited ability to broadly protect populations over a long duration but the public health benefits were important in vaccine implementation decisions in those countries (Wilder-Smith et al., 2017). This suggests a paradigm for alternative regulatory requirements with a focus on public health outcomes (Gessner et al., 2017).

### Cost-Effective Preparedness for Outbreaks

As human populations grow and their use of the finite land resources increases, we are in increasingly close association with other living creatures, voluntarily or involuntarily. This interaction with natural reservoirs of potential infectious diseases increases the risk of zoonotic transmission of new infectious pathogens e.g., SARS, MERS-CoV, or known infectious pathogens with increased virulence e.g., influenza. Emerging infectious diseases disproportionately affect developing regions, where health infrastructure and surveillance are likely to be less well-established and robust. There were 1,307 epidemics of infectious diseases between 2011 and 2017, which cumulatively cost $60 billion annually to manage (GHRF Commission, 2016). The unpredictability of outbreaks was highlighted by the Ebola epidemic in Western African countries of Liberia, Sierra Leone, and Guinea in 2014, which occurred in a period when public health was supposedly at its most advanced in recent history. However, a catalog of areas including: outbreak planning infrastructure; disease surveillance; local health services; escalation to international agencies were found to be lacking (GHRF Commission, 2016). Although the WHO received criticism for its lack of escalation, in reality the global and interconnected infrastructure to prevent such epidemics taking lives and devastating societies is insufficient at the present time. The Zika virus epidemic in Latin America in 2015, first observed through an unexpectedly high incidence of microcephaly amongst newborns in Brazil’s northern regions (Heukelbach et al., 2016), provide another example of how epidemics can have lasting impact, with the virus causing significant neurological damage to surviving infants (Russo et al., 2017). The SARS-CoV-2 pandemic which began in 2019, was, at the time of writing, the largest infectious disease pandemic since the influenza pandemic of 1918/9. This global public health crisis highlighted stark societal inequalities persistent in many high-, middle- and low-income countries with direct and indirect impact on health outcomes from this infection. The cost-effectiveness of a vaccine in this setting was unquestionable, with economies and societies shut down for months in early 2020 and likely again in future. As it is not feasible or practical to be able to predict the location or nature of the next emerging threat, investment of an estimated $4.5 billion/year in healthcare systems could help speed up responses to infectious epidemics by prompt identification of the agent and effective control measures to limit the spread and consequences of disease (GHRF Commission, 2016). The importance of this planning within the political landscape and the ongoing threat that infectious disease pose, may be appreciated more widely after 2020.

### Establishing Programs for Vaccine Development

One effective infection control method is the use of vaccines in the course of an epidemic to halt transmission and to induce immunity to those as yet unaffected. The cost of vaccine development is a major challenge as there is little incentive for industry to invest in the design, testing and manufacture of vaccines that may never be needed, have a limited market, and, as previously eluded to, may be required in LMIC which cannot afford the upfront costs as an epidemic unfolds. The estimated costs for funding the development of infectious diseases vaccines for epidemics through phase 2a clinical trials are a minimum of $2.8-3.7 billion (Gouglas et al., 2018). The CEPI alliance was established at the Davos World Economic Forum in 2017 as a global partnership between public, private and philanthropic organizations. In response to the conclusion that “a coordinated, international, and intergovernmental plan was needed to develop and deploy new vaccines to prevent future epidemics,” CEPI have identified the most important known global infectious threats and invested in the development of vaccines, stockpiling, and policies to allow equitable access to these (Plotkin, 2017). Further, the establishment of research and development infrastructure pipelines will allow production of suitable vaccine candidates within 16 weeks of identification of a new pathogen antigen. The broader aims including: improving global epidemic responses; capacity building; and global regulation of outbreak management strategies are also within the remit of CEPI’s work. It is these types of preparedness plans that assisted vaccine development and global health collaborations to address the COVID-19 pandemic, though many regions of high-, middle-, and low-income countries alike were slow or resistant to pre-empt and prepare for this type of infectious disease threat.

## Social Benefits

### Equity of Healthcare

As a result of the combined effects of poverty, malnutrition, poor hygiene and sanitation, overcrowding, discrimination and poorer access to health-care, the underprivileged in society are disproportionately afflicted by infectious diseases. Over the 20th century, it has become a moral standpoint and a human right for every individual to be provided with access to safe vaccines. The provision of vaccination as part of the EPI on a national and international scale (World Health Assembly, 1974) acted as a great leveler to start reducing the impact of infectious diseases to all, regardless of other disadvantages. Over the 15 years of the EPI, the vaccine coverage in developing countries increased from 5% to ∼80% (Levine and Robins-Browne, 2009). The EPI was revolutionary for its time, an ambitious public health program that aimed to improve children’s life chances despite the country and situation in which they were born. The administration of vaccines by UNICEF was deemed so important that there have been at least seven ceasefires in civil conflicts to allow this to happen (Hotez, 2001).

The impact of vaccines on the inequity of those living in poverty is marked. A study of over 16,000 children during the phased introduction of the measles vaccine in Bangladesh in 1982, demonstrated improved health outcome equity when measured by under-5 mortality (Bishai et al., 2003). Further, modeling of the impact of the rotavirus vaccine in India across social strata, which are closely aligned to wealth, suggested that the vaccine program would provide the poor with both health and financial benefits (Verguet et al., 2013). Including such equity impact in the health economic modeling of vaccines would allow policy decisions to be targeted to the most vulnerable in society (Riumallo-Herl et al., 2018). Additional cost-effective benefits observed after the implementation of combined public health initiatives (Deogaonkar et al., 2012; Gessner et al., 2017) include provision of vaccines, facilitation of healthcare, reduction of indoor air pollution and improvement of nutrition to prevent childhood pneumonia (Niessen et al., 2009).

### Strengthening Health and Social Care Infrastructure

To provide the EPI universally to infants and children, a significant degree of healthcare infrastructure is required ranging from primary care to public health. An example of the multiple facets of a successful vaccine program were outlined in the Mission Indradhanush in India, which planned to make life-saving vaccines available to all children and pregnant women by 2020 through programs with (i) national, (ii) state, (iii) district, and (iv) block/urban level input (Hinman and McKinlay, 2015). National programs require governments to provide financial resources and set out policy for implementation. States needed to obtain the vaccines and to store them appropriately whilst eligible children were identified through public health messaging and outreach. Districts and urban areas recruited staff trained in vaccine delivery and communication to administer vaccines and to provide the aftercare where required. Establishing this degree of nationwide infrastructure to reach those in urban and rural areas, provides the basis for the provision of other health and social care services for all members of the community, in particular improving maternal and infant mortality in developing regions and in the elderly in industrialized regions (Shearley, 1999). Public health infrastructure and personnel could be used to promote other important messages and health education (Shearley, 1999), relating to malnutrition, hygiene and sanitation and preventable diseases such as malaria and HIV infection. Global drivers are also key, as demonstrated by the establishment of the EPI in 1974, when all countries were directed to provide these vaccines, thereby developing their primary- and public health-care infrastructure, with benefit beyond the vaccine program. Vaccination contributes to the UN Millennium Development Goals and later Sustainable Development Goals for achievement by 2030. Gavi, the Vaccine Alliance, has been an important provider of funds, vaccines and support for countries whose gross national income per capita was <£1000/year (Hinman and McKinlay, 2015). The partnerships forged through the development of vaccine programs in LMIC, can be long-lasting and beneficial through other health and social care endeavors (Shearley, 1999).

### Impact of Life Expectancy and Opportunity

Vaccination programs provide a degree of social mobility, as poverty and the associated ill-health and mortality from infectious diseases are no longer the determinants of one’s life chances. Vaccine recipients have the potential for improved life-expectancy largely demonstrated by, but not confined to, infants and children (Andre et al., 2008). It has become increasingly recognized that an aging population goes through the process of immunosenescence (Fulop et al., 2017), and increased incidence and severity of infectious diseases. In many countries, therefore, older people are offered vaccines to prevent infections with high mortality and morbidity, including the influenza, pneumococcal, herpes zoster, and pertussis vaccines (Bonanni et al., 2015). These prevent the development of pneumonia, admission to hospital and the subsequent associated risks of death from cardiac failure, as observed in Sweden (Christenson et al., 2004).

The global and interconnected world of the 21st century provides opportunity to discover new cultures, new environments and their resident microbes. The safety of global travel has been greatly enhanced by the availability of vaccines that provide protection against organisms that are different to those in a person’s home setting. Movement of people may be through necessity when fleeing war and conflict, in the search of better life opportunities, or for leisure purposes. For mass movements of refugees vaccines are crucial to the aid and relief efforts to support these individuals (Hermans et al., 2017), as measles and cholera can be highly problematic in refugee camps. Global mass cultural or religious gatherings, such as the Hajj pilgrimage (Yezli et al., 2018) or the Chinese New Year (Chen et al., 2018) have been implicated in the spread of meningococcal disease outbreaks. Pre-travel vaccines offer the optimal level of protection for those with scheduled travel plans and include protection against: yellow fever, hepatitis A and B, rabies, Japanese encephalitis, tick-borne encephalitis, typhoid, and cholera.

### Empowerment of Women

The empowerment of women is both a driver and effect of vaccination programs. The degree of education, literacy and independence of girls and women varies considerably across the world and within countries. Where women have the information and autonomy to make health-related decision for their children, childhood immunization rates improve. In a study in Bihar State in rural India involving an empowerment program, where participating women were educated about health and hygiene, there was a higher rate of DTP, measles and BCG vaccination in their children compared to the non-participants in the villages running the program (Janssens, 2011). Further, this information and autonomy served to improve the rates of vaccination in children of non-participants in the villages running the program compared to control villages not running the education program, through social or formal ongoing dialogue within the village community. A separate public health initiative in Haryana, India conducted between 2005 and 2012 to reduce maternal and child health inequalities, involved improving access and provision of health resources to rural areas, the poor in society, women and children. One significant outcome of this initiative was the equitable provision of immunizations to girls and boys, despite the male-favored disparity prior to starting the public health initiative (Gupta et al., 2016).

By improving infant and childhood mortality from infection, more children will survive to adulthood with the potential to have productive and healthy lives. This has led to healthy and economically secure women having fewer children and less peripartum morbidity and mortality (Sah, 1991; Shearley, 1999). Thus, women are able to spend more time with their children and on their development (Shearley, 1999) as well as their own education and contribution to the workforce. The strategy of maternal vaccination has demonstrated great success at preventing diseases that afflict infants too young to be vaccinated against pertussis, influenza and tetanus (Marchant et al., 2017). Factors influencing the uptake of maternal vaccination include women’s previous experiences with healthcare and vaccines, so it is crucial to provide the access and support required to enable them to make informed choices during their pregnancy (Wilson et al., 2019).

## Conclusion

The impact of vaccines is broad and far-reaching, though not consistently quantifiable, analyzed or communicated. Traditionally, the perceived benefits of vaccination were to reduce morbidity and mortality from infections, and those remain the drivers for the innovation of new vaccines, in particular in preparation for outbreaks or against infections that afflict the most disadvantaged in society. However, an increasing appreciation for the economic and social effects of vaccines is being included in the development and assessment of vaccine programs, potentially realizing a greater benefit to society and resulting in wider implementation. There remain challenges to delivering vaccines to all children and vulnerable people worldwide, in particular those in communities that are difficult to reach geographically, politically and culturally and these challenges can only be overcome with the continued commitment and dedication to this endeavor on an international, national and individual scale.

## Author Contributions

SP conceptualized and designed the study. CR prepared the manuscript and figures. CR and SP contributed to literature search and revision and review of the final manuscript. Both authors contributed to the article and approved the submitted version.

## Conflict of Interest

SP consults for many major vaccine manufacturers and biotechnology companies but this article was unfunded.

The remaining author declares that the research was conducted in the absence of any commercial or financial relationships that could be construed as a potential conflict of interest.
